# A New Tetracyclic Bromopyrrole-Imidazole Derivative through Direct Chemical Diversification of Substances Present in Natural Product Extract from Marine Sponge *Petrosia* (*Strongylophora*) sp.

**DOI:** 10.3390/molecules28010143

**Published:** 2022-12-24

**Authors:** Natchanun Sirimangkalakitti, Kazuo Harada, Makito Yamada, Masayoshi Arai, Mitsuhiro Arisawa

**Affiliations:** Graduate School of Pharmaceutical Sciences, Osaka University, 1–6 Yamadaoka, Suita 565-0871, Osaka, Japan

**Keywords:** natural product extract, natural product-like compound, chemical diversification, one-step reaction, marine sponge *Petrosia* (*Strongylophora*) sp., bromopyrrole imidazole alkaloid, cylindradines

## Abstract

Chemical diversification of substances present in natural product extracts can lead to a number of natural product-like compounds with a better chance of desirable bioactivities. The aim of this work was to discover unprecedented chemical conversion and produce new compounds through a one-step reaction of substances present in the extracts of marine sponges. In this report, a new unnatural tetracyclic bromopyrrole-imidazole derivative, *rac*-6-*O*Et-cylindradine A (**1**), was created from a chemically diversified extract of the sponge *Petrosia (Strongylophora)* sp. We also confirmed that **1** originated from naturally occurring (-)-cylindradine A (**2**) via a new reaction pattern. Moreover, (-)-dibromophakellin (**3**) and 4,5-dibromopyrrole-2-carboxylic acid (**4**), as well as **2**, were reported herein for the first time in this genus. Studies on the possible reaction mechanism and bioactivities were also conducted. The results indicate that the direct chemical diversification of substances present in natural product extracts can be a speedy and useful strategy for the discovery of new compounds.

## 1. Introduction

Natural products (NPs) are considered to be significant sources of novel scaffolds and bioactive molecules. According to recent studies, approximately half of all approved drugs for the treatment of human diseases are based on NPs [1]. Additionally, over 100 NPs and NP-derived compounds have been used in clinical trials or in registration for various diseases, particularly as anticancer and anti-infective agents [2]. NPs exhibit considerable structural complexity and diversity that differs from synthetic small-molecule libraries in different aspects, such as higher molecular mass, larger number of *sp*^3^ carbon and oxygen atoms, higher hydrophilicity, and greater molecular rigidity [3]. Although the successful records and advantages of NPs in drug discovery are widely recognized, NP research has largely scaled back from the pharmaceutical industry. To further drug discovery research, humanity needs a more diverse group of compounds in terms of chemical space. Several research groups have developed strategies to explore novel compounds with desirable biological activities [4]. One of the most promising approaches is direct chemical diversification of substances present in NP extract, with mostly uncharacterized composition, by a simple chemical reaction to reach a number of NP-like compounds in one step. Previously, the studies using this concept have been reported. The chemical reactions, such as ammonolysis [5,6], oxidation [7,8], sulfonylation [9], bromination [10], and fluorination [11], could transform chemical groups that are highly common in NPs to chemical groups rarely found in nature. This can be applied to various types of compounds for modifying chemical groups in NPs and even remodeling molecular skeletons [12,13,14]. Although the previous research provided successful results, there are currently not enough methodologies for chemical diversification of substances present in NP extracts.

**Figure 1:**
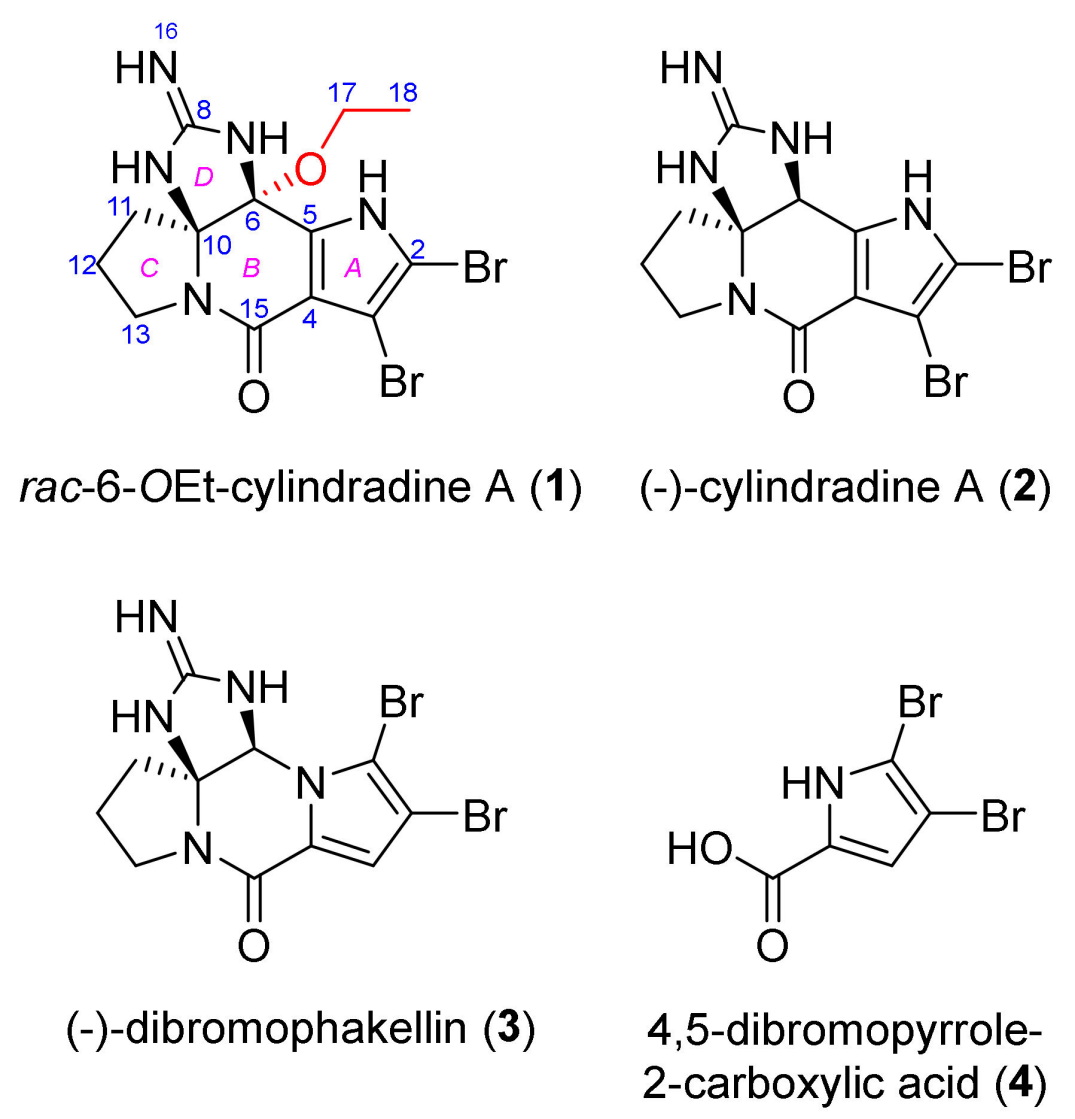
The chemical structures of a new unnatural derivative of alkaloid (**1**) and naturally occurring compounds (**2**–**4**).

The aim of this research is to discover unprecedented chemical conversion and produce new compounds through a one-step reaction of substances present in the extracts of marine organisms. Marine sources have been receiving increasing attention, as they can produce chemically novel bioactive metabolites [15,16]. As part of our ongoing research on the chemical diversification of substances present in several marine NP extracts from sponges and sponge-derived fungi using various heat-treated solvents, including EtOH, 1,4-dioxane, acetonitrile, and *p*-xylene, we found one successive reaction of the substance present in the extract from the marine sponge *Petrosia* (*Strongylophora*) sp. (Petrosiidae, Haplosclerida). The marine sponges of the genus *Petrosia* contain a variety of bioactive metabolites. Of the isolated compounds reported between 1978 and 2020, polyacetylenes (53%), meroterpenoids (19%), and sterols (16%) were the most frequently found, while alkaloids (6%), fatty acids (3%), peptides (2%), and saponins (1%) were not very common [17]. In this paper, we report the successful novel chemical diversification of the substances present in the extract of the marine sponge by using heat-treated EtOH. Introduction of ethoxy into a natural molecule led to the discovery of a new, unexpected derivative of alkaloid via a new reaction pattern, which differs from previous ones. Isolation, structure elucidation, studies of possible reaction mechanisms, and bioactivity evaluation of a new unnatural derivative of alkaloid together with naturally occurring compounds (Figure 1) were described.

## 2. Results and Discussion

### 2.1. Chemical Diversification of Substances Present in NP Extract and Reversed-Phase Liquid Chromatography/Mass Spectrometry (RP-LCMS) Detection

**Figure 2:**
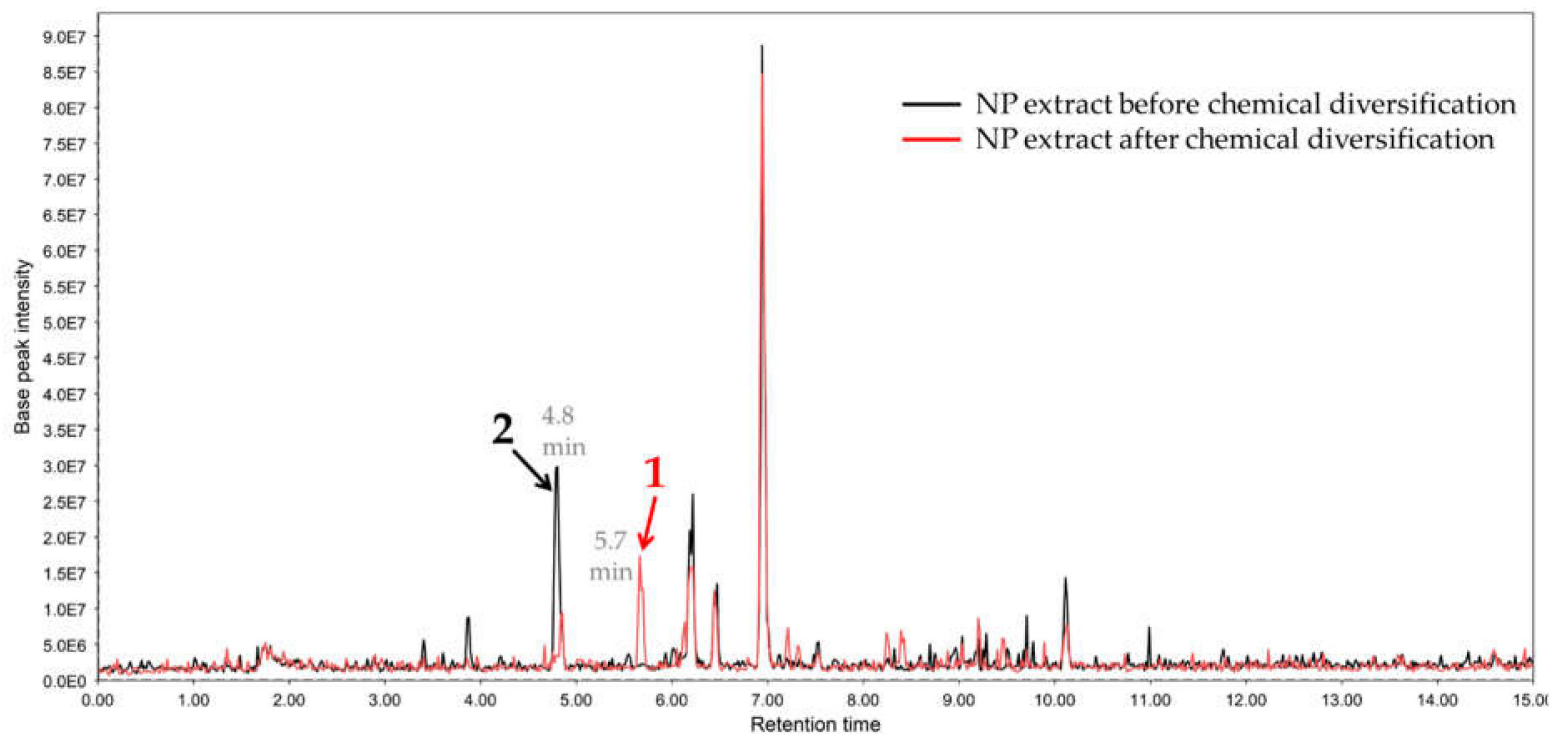
Base peak chromatogram (positive ion mode, reversed-phase liquid chromatography/mass spectrometry (RP-LCMS)) of natural product (NP) extracts before (black line) and after chemical diversification (red line). The retention time is shown on the x-axis, going from 0 to 15 min, and the base peak intensity is shown on the y-axis, going from 0 to 9.0 × 10^7^. Peaks of *rac*-6-*O*Et-cylindradine A (**1**) at retention time (RT) 5.7 min and (-)-cylindradine A (**2**) at RT 4.8 min are pointed out by red and black arrows, respectively.

The marine sponge *Petrosia* (*Strongylophora*) sp. (dry weight 50 g) was extracted using MeOH (400 mL × 7 times) at room temperature to obtain a crude MeOH extract (7 g). The crude MeOH extract was subjected to prefractionation using reversed-phase medium pressure liquid chromatography (RP-MPLC) which was eluted with 30%, 50%, 70%, 80%, and 100% MeOH in H_2_O to provide each corresponding fraction (2.5, 0.2, 0.2, 0.1, and 0.3 g, respectively). Prefractionation of the crude MeOH extracts could be useful to reduce the complexity of the diversified extracts and to the following chemical reactions due to better solubility. A portion of each fraction was dissolved in either EtOH, 1,4-dioxane, acetonitrile, or *p*-xylene (conc. 1 mg/3 mL) and heated at 80, 105, 85, and 140 °C, respectively, under N_2_ atmosphere. After 72 h, the reaction mixtures were concentrated under reduced pressure to obtain diversity-enhanced extracts. Comparison of RP-LCMS chromatograms before and after the chemical diversification showed interesting changes in the reaction mixture of the 50%MeOH fraction with heat-treated EtOH (Figure 2). The reaction was repeated to check whether there is no problem with reproducibility. A peak at retention time (RT) 5.7 min of *rac*-6-*O*Et-cylindradine A (**1**, *m*/*z* 432 [M + H]^+^, 434, 436) could be detected, whereas a peak at RT 4.8 min of (-)-cylindradine A (**2**, *m*/*z* 388 [M + H]^+^, 390, 392) significantly decreased after the reaction. This finding raised the possibility that **2** was converted into **1**. Moreover, the molecular mass of **1** was 44 mass units larger than that of **2**, corresponding to the introduction of ethoxy into a natural molecule through the exchange of one hydrogen atom.

**Scheme 1:**
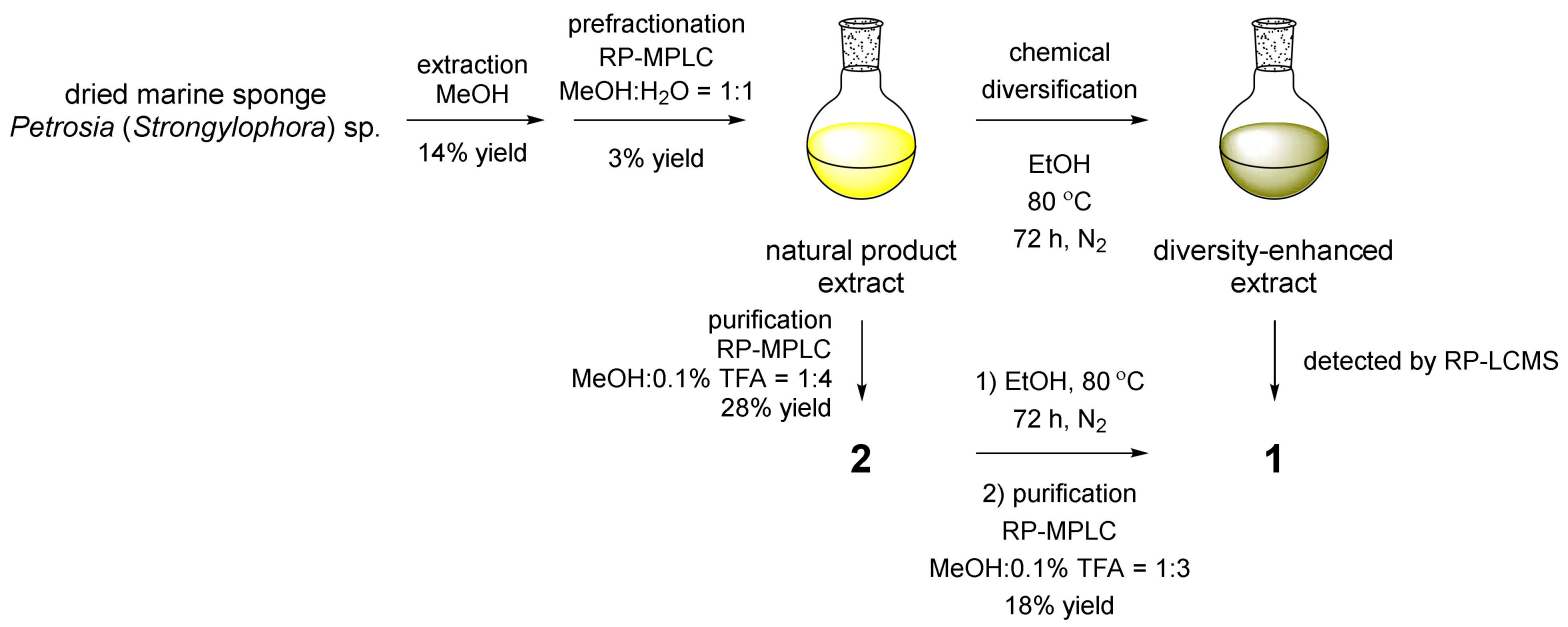
*rac*-6-*O*Et-cylindradine A (**1**), was detected in a diversity-enhanced extract after direct chemical diversification of the substances present in the natural product extract from marine sponge *Petrosia* (*Strongylophora*) sp. Then, it was confirmed that **1** originated from (-)-cylindradine A (**2**). Abbreviations: RP-MPLC, reversed-phase medium pressure liquid chromatography; TFA, trifluoroacetic acid.

To obtain sufficient quantity to determine the structure of **1** and confirm the possibility that **1** was converted from **2** after the chemical reaction, a larger amount of the marine sponge (dry weight 203 g) was extracted using MeOH to obtain a crude MeOH extract (28 g), and then prefractionation by the same procedure. Purification of the 50% MeOH fraction (0.8 g) by RP-MPLC which was eluted with 20% MeOH in 0.1% trifluoroacetic acid (TFA)·H_2_O provided **2** (221 mg, 28% yield), then half of the amount of **2** was submitted to chemical treatment as described above. The reaction mixture (106.0 mg) was purified by RP-MPLC which was eluted with 25% MeOH in 0.1% TFA·H_2_O to obtain **1** (19.1 mg, 18% yield). This result certainly confirmed that the new unnatural derivative of alkaloid **1** originated from **2** (Scheme 1).

### 2.2. Structure Elucidation

*rac*-6-*O*Et-cylindradine A (**1**) was obtained as an amorphous solid, m.p. 243 °C (decomp.). The RP-LCMS displayed pseudomolecular ion peaks at *m*/*z* 432, 434, 436 (1:2:1), suggesting the existence of two bromine atoms in the molecule. The high-resolution MALDI mass spectrometry (HRMALDIMS) revealed the molecular formula as C_13_H_16_^79^Br_2_N_5_O_2_ (*m*/*z* 431.9656 [M + H]^+^, calcd 431.9665). The ultraviolet (UV) absorption bands at lambda max (λ_max_) 248, 273 nm of **1** were almost identical to that of **2** (λ_max_ 244, 270 nm) [18], indicating that they possess the same pyrrole ring conjugated with a carbonyl group. The infrared (IR) absorption bands (MeOH) exhibited at 1663 (carbonyl group and C=N bond), and 3200, 3410 (amino group) cm^−1^. The ^1^H and ^13^C nuclear magnetic resonance (NMR) spectra data and HMBC correlation of **1** in DMSO-*d*_6_ are shown in Table 1. The ^13^C NMR spectrum disclosed thirteen carbons, consisting of an amide carbonyl (*δ*_C_ 157.2), five *sp*^2^ quaternary carbons (*δ*_C_ 156.0, 131.5, 114.0, 106.6, 95.5), two *sp*^3^ quaternary carbons (*δ*_C_ 86.7, 86.0), four methylenes (*δ*_C_ 61.3, 44.3, 34.3, 19.2), and a methyl carbon (*δ*_C_ 14.8). By comparing ^1^H and ^13^C NMR data of **1** with those of **2**, it was suggested that they possessed the same skeleton that has a 3-carbamoylpyrrole ring A, a pyrrolidine ring C, and an imidazoline ring D [18]. However, the ^13^C NMR of **1** differed from that of **2** by the signal of C-6, which shifted to downfield due to the connection of oxygen, as well as the presence of a methylene carbon and a methyl carbon at *δ*_C_ 61.3 and 14.8, respectively. In addition, the methine proton (*δ*_H_ 5.28, H-6) in **2** was not observed in **1**.

**Figure 3:**
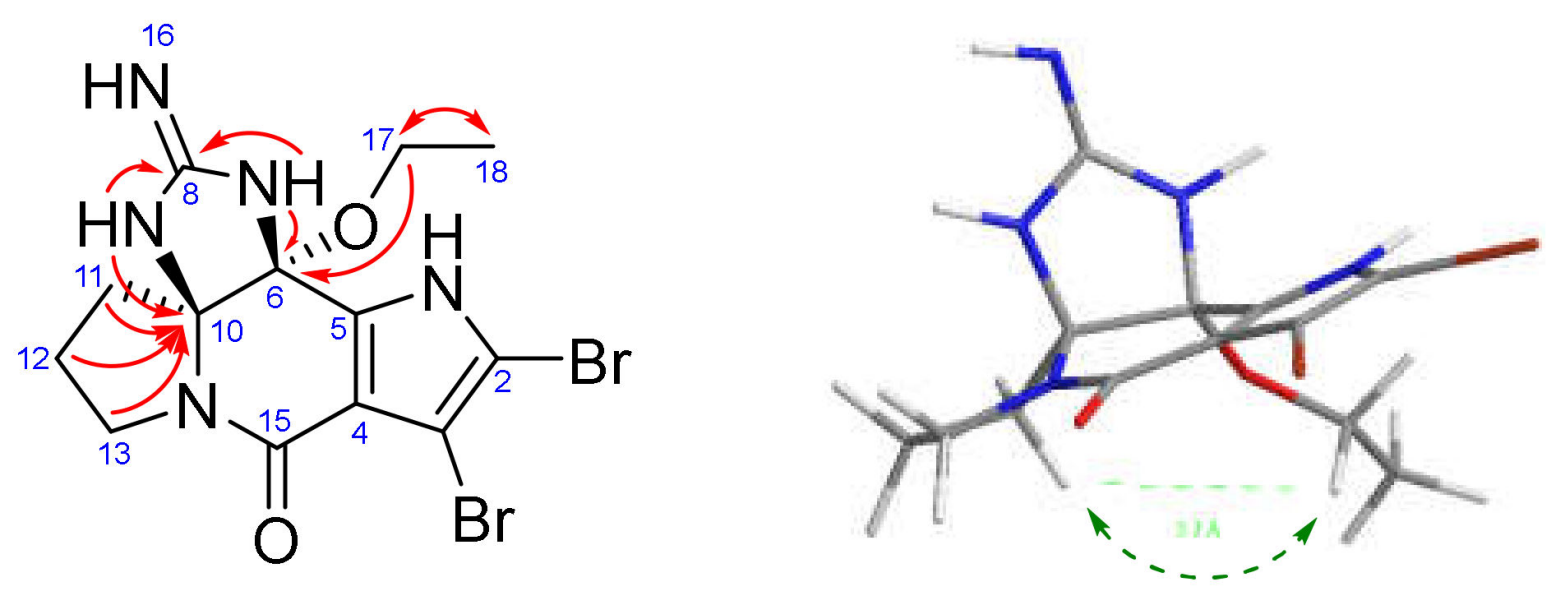
Key HMBC (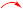) and 2D-NOESY (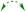) correlations of *rac*-6-*O*Et-cylindradine A (**1**).

The structure of **1** was assigned by detailed analyses of ^1^H-^1^H COSY, HSQC, and HMBC. The HMBC correlations for H-17 (*δ*_H_ 3.29, 3.38)/C-6 (*δ*_C_ 86.7) supported an ethoxy substituted at ring junction C-6 (Figure 3). Furthermore, the *cis* fusion of the imidazoline moiety at C-6 and C-10 positions, as shown in Figure 3, was suggested by the 2D-NOESY cross peak between H-11 and H-17. The specific rotation of **2** was determined to be ${\left[\alpha \right]}_{D}^{20}$ −76.7 (c 0.22, MeOH), suggesting that **2** was isolated as a mixture of enantiomers (a ratio 7:3) [18], but on the other hand, the specific rotation of **1** was optically inactive (${\left[\alpha \right]}_{D}^{27}$ +0.01 (c 0.50, MeOH)). A chiral high pressure liquid chromatography (HPLC) analysis (CHIRALPAK^®^ IE, hexane:EtOH:diethylamine = 85:15:0.1) revealed that **1** was a racemic mixture (Supplementary Materials Figure S11).

Furthermore, isolation of the 50% MeOH fraction by RP-MPLC which was eluted with 20% and 90% MeOH in 0.1%TFA·H_2_O also gave two known bromopyrrole derivatives, (-)-dibromophakellin (**3**) and 4,5-dibromopyrrole-2-carboxylic acid (**4**), respectively. The yields of compounds **2**, **3**, and **4** were approximately 0.1%, 0.01%, and 0.2% sponge dry weight, respectively. The structures of compounds **2**-**4** were assured by comparing their NMR data with the literature [18,19,20]. Compound **2** was previously isolated solely from the sponge *Axinella cylindratus* (Axinellidae, Axinellida) [18], while **3** and **4** were first isolated from *Phakellia flabellate* (Axinellidae, Axinellida) [21] and *Agelas oroides* (Agelasidae, Agelasida) [22], respectively, in 1971. The naturally occurring **2**-**4** were reported herein for the first time in the genus *Petrosia* (*Strongylophora*).

### 2.3. Experiments to Investigate the Possible Reaction Mechanisms

**Scheme 2:**
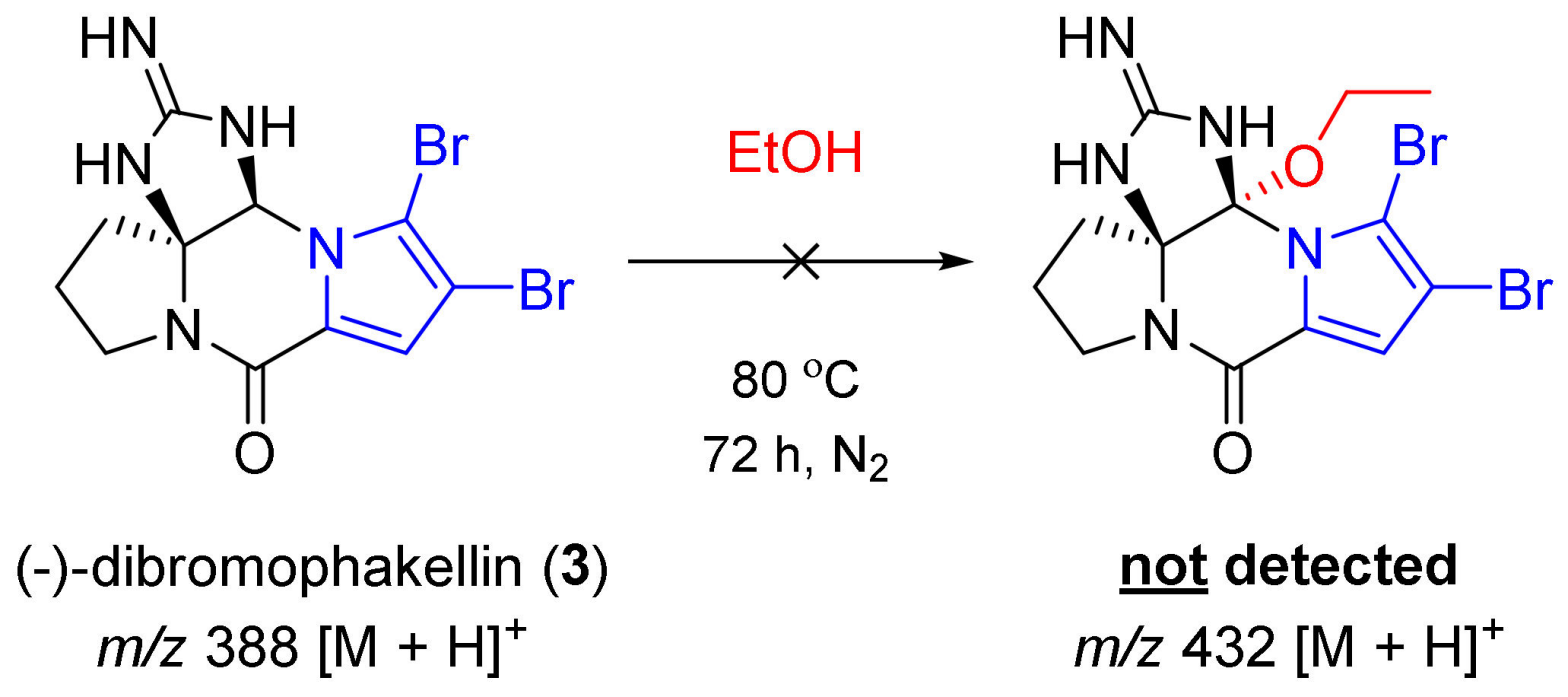
Heating (-)-dibromophakellin (**3**) with EtOH. Mass detection of reaction products was analyzed by RP-LCMS.

We conducted experiments to gain insight into possible reaction mechanisms. As expected, no reaction was observed by RP-LCMS upon heating **3** with EtOH (Scheme 2). This indicates a nitrogen of 3-carbamoylpyrrole ring A might be responsible for the introduction of the angular ethoxy group at C-6. EtOH acts as a nucleophile that displaces a hydrogen atom in **2** to form the product **1**. We also confirmed that compound **4** did not react with EtOH.

To clarify whether the reaction proceeds through a radical mechanism, the radical scavenger galvinoxyl was introduced in the reaction of **2** in a stoichiometric amount; however, galvinoxyl did not affect the product yield of the reaction (same base peak intensity of **1** between reactions with and without galvinoxyl, Figure S12). Our reaction seems unlikely to involve radicals.

In addition, the reactions of **2** with EtOH were carried out at 25, 50, and 80 °C. The results showed that no production of **1** was observed at 25 °C, whereas the reaction at 50 °C yielded **1** in a lower amount compared to that from the reaction at 80 °C (Figure S13). It indicated that the preparation of **1** was markedly sensitive to temperature.

**Scheme 3:**
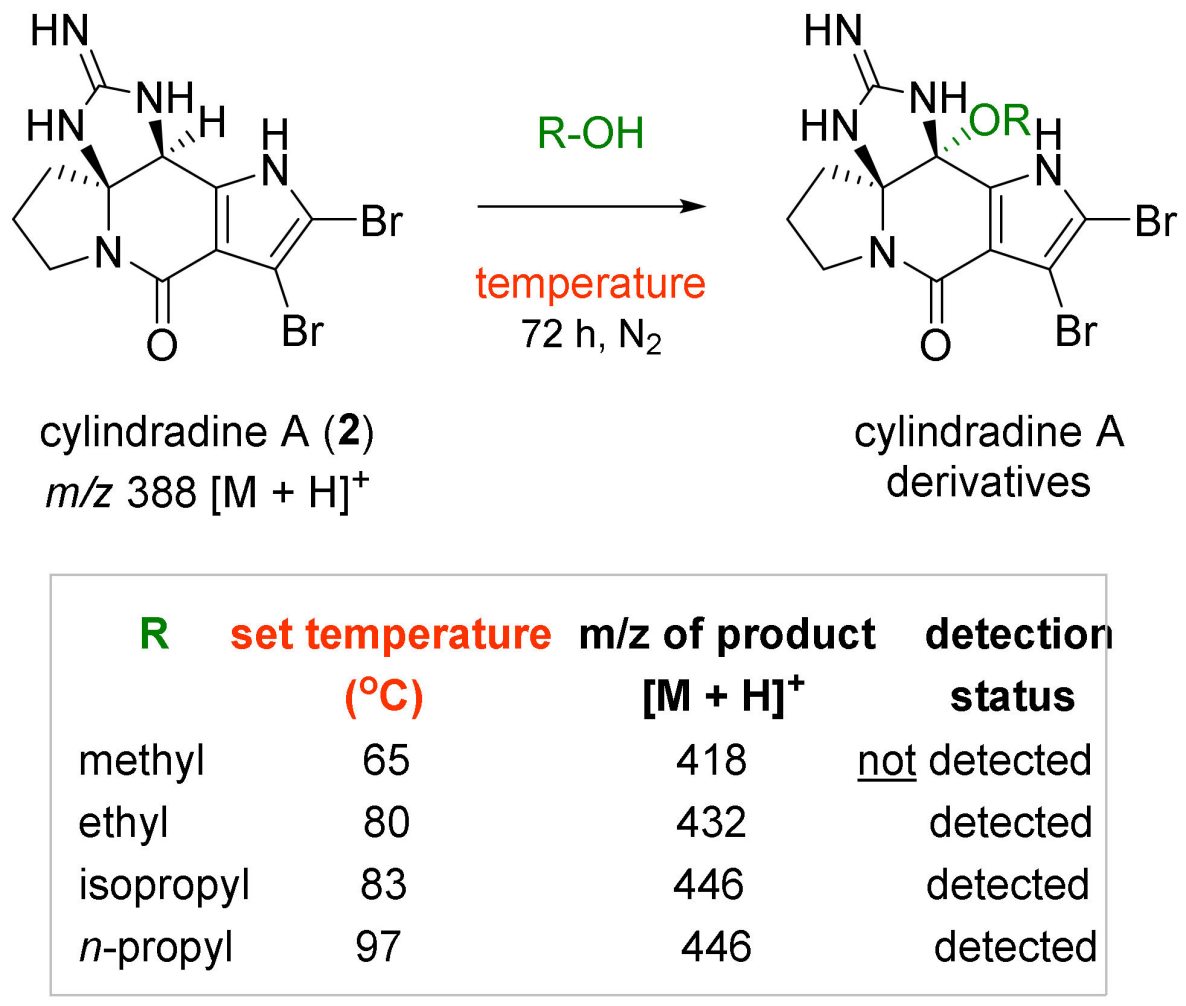
Chemical conversion of (-)-cylindradine A (**2**) with various aliphatic alcohols at their corresponding boiling temperatures. Mass detection of reaction products was analyzed by RP-LCMS.

Chemical conversions of **2** with various aliphatic alcohols were conducted (Scheme 3). Treatment of **2** with MeOH at its boiling point (65 °C) did not lead to the formation of 6-*O*Me-cylindradine A, whereas treating **2** with isopropanol and *n*-propanol at higher temperatures (boiling points 83 °C and 97 °C, respectively) led to the introduction of a propoxy group into the structure, which could be detected as *m*/*z* 446 [M + H]^+^ using RP-LCMS. We believe that it is due to the appropriate reaction temperature.

### 2.4. Biological Activities

Pyrrole-imidazole alkaloids are a group of intriguing NPs with a range of significant pharmacological and ecological bioactivities, such as anticancer, antimicrobial, and feeding deterrent activities [23]. Compound **2** was reported as a moderate inhibitor against the P388 murine leukemia cell line (50% inhibitory concentration (IC_50_) 20 μM) [18]. In our study, all four compounds were evaluated for anticancer activity against four cancer cell lines, and antimycobacterial activities (Table 2). The changes in chemical structure were expected to translate into changes in biological activities; however, **1** and **2** were not active against any of the tested cancer cells or mycobacteria (IC_50_ > 100 μM for anticancer activity; minimum inhibitory concentration (MIC) > 100 μM for antimycobacterial activity). Due to rare type structure, scarcity in natural sources, stability, and a challenging total synthesis of **2**, there have limited studies to evaluate its bioactive potential [24]. Further investigation in biological activities remains to be explored.

### 2.5. Discussion: Further Consideration of Our Finding

For the purpose of drug discovery, direct chemical diversification of substances present in NP extracts is an effective methodology for producing new compounds. It is in the early stages and rapidly progressing. The dramatic improvements in speed of discovery of new compounds might allow NP research to be more competitive with synthetic compound screening. This approach is also essential to retain the usefulness of NPs and their derivatives in pharmaceutical research; however, it remains an extremely challenging task due to the high composition complexity with several hundreds of compounds. Unexpected side reactions and the decomposition of substances present in NP extract cause complications in the reaction system, resulting in difficulties identifying the products from chemically diversified extracts. In addition, we suggest that marine-derived organisms can serve as good candidates for direct chemical diversification of substances present in NP extracts [25,26,27]. In order to expand the chemical diversity of marine NP extracts, other types of reactions should be applied.

## 3. Materials and Methods

### 3.1. General Experimental Procedures

NMR spectra were measured on a Varian Inova 600-II NMR spectrometer (Varian, Inc., CA, USA). Chemical shifts were referenced to the residue solvent peaks: DMSO-*d*_6_ (δ_H/C_ 2.50/39.5) and CD_3_OD (δ_H/C_ 3.31/49.0). HRMALDIMS spectra were recorded on a SpiralTOF™ JEOL JMS-S3000 mass spectrometer (JEOL Ltd., Tokyo, Japan). A melting point was determined on a Buchi B-545 apparatus (Flawil, Switzerland) or MP-J3 micro melting point apparatus (Anatec Yanaco Corp., Kyoto, Japan). IR spectra were recorded on an IRAffinity-1 or IRAffinity-1S (Shimadzu Corporation, Kyoto, Japan). Optical rotations were measured on a Jasco P-1020 polarimeter (JASCO Corporation, Tokyo, Japan) in MeOH. RP-LCMS experiments were performed on an Acquity UPLC system coupled to a Quattro Premier XE mass spectrometer fitted with an electrospray ionization (ESI) interface. Separations were performed on an Acquity UPLC BEH C18, 2.1 × 150 mm, 130 Å, 1.7 μm (Waters Corporation, MA, USA). The mobile phase was composed of MeCN and 0.1% HCOOH·H_2_O. The flow rate was 0.25 mL/min at 40 °C, and the injections were carried out through a 5 μL-loop. UV data were collected on a UV photodiode array detector. The RP-LCMS data was processed using a MZmine 2.53 (MZmine Development team). RP-MPLC was conducted using a dual channel automated Smart Flash EPCLC-W-Prep 2XY system (Yamazen Corporation, Osaka, Japan). The medium-pressure ODS (C18) chromatographic column (Yamazen Corporation, universal column size L: 3.0 × 16.5 cm; M: 2.3 × 12.3 cm; S: 1.8 × 11.4 cm, pore size 120 Å, particle size 30 or 50 μM) was conditioned by first eluting with 100% MeOH or MeCN, then equilibrating with a suitable initial mobile phase. After dissolving the extract in the initial mobile phase, the solution was loaded in the ODS (C18) inject column (Yamazen Corporation, size M: 2.0 × 7.5 cm; S: 1.5 × 4.4 cm; SS: 1.3 × 3.1 cm) and separated by the gradient elution program. The UV wavelength detection was at 230 nm. The fractions were collected automatically based on time. The purity of each collected fraction was determined by analytical reversed-phase high pressure liquid chromatography (RP-HPLC). When the purity of the target compound was less than 90%, it was submitted to further purification. An analytical RP-HPLC system was composed of a LC-20AD pump, a DGU-20A3R degasser, a CTO-20AC column oven, and an SPD-M20A diode array detector (Shimadzu Corporation, Kyoto, Japan). Separations were performed on a Xbridge^®^ C18 (4.6 × 150 mm, 130Å, 3.5 μm) coupled with a XBridge^®^ BEH C18 Vanguard^®^ Cartridge (3.9 × 5 mm, 130 Å, 3.5 µm, Waters Corporation), unless otherwise explained. The mobile phase was composed of either MeOH or MeCN and 0.1% TFA·H_2_O degassed by sonication. The flow rate was 0.5 mL/min at 25 °C, and the injections were carried out through a 20 μL-loop. Data analysis was performed by Labsolutions (Shimadzu Corporation).

### 3.2. Sponge Material

The marine sponge *Petrosia* (*Strongylophora*) sp. was collected by SCUBA divers at a depth of 10–15 m at Lembeh Island, Indonesia in August 1999. It was cut into small pieces and air dried at the collecting place. The sponge was identified by Dr. Nicole J. de Voogd. The voucher specimens have been deposited at the National Museum of Natural History, Leiden, The Netherlands, and the Laboratory of Natural Products for Drug Discovery, Graduate School of Pharmaceutical Sciences, Osaka University (under the code number 9930H12).

### 3.3. Extraction and Prefractionation of the MeOH Crude Extract

The sponge (dry weight 50 g) was macerated in MeOH (400 mL × 7 times) at room temperature. After maceration, the solution was filtered and evaporated to dryness on a rotatory vacuum evaporator to obtain a crude MeOH extract (7 g). Prefractionation of the MeOH extract using RP-MPLC which was eluted with 30%, 50%, 70%, 80%, and 100% MeOH in H_2_O provided each corresponding fraction (2.5, 0.2, 0.2, 0.1, and 0.3 g, respectively). The 30%, 50%, 70%, 80%, and 100% MeOH fractions were used for chemical diversification.

### 3.4. Chemical Diversification of Substances Present in NP Extracts to Prepare the Diversity-Enhanced Extracts

Initially, 1 mg of the obtained fractions was transferred to 15 mL test tubes (15 × 150 mm), and then dissolved in 3 mL of either EtOH, 1,4-dioxane, acetonitrile, or *p*-xylene. The test tubes were placed in the ChemiStation PPM-5512 apparatus (EYELA, Tokyo, Japan) whose temperature setting was stabilized. Temperatures were set to 80, 105, 85, and 140 °C for EtOH, 1,4-dioxane, acetonitrile, and *p*-xylene, respectively. After 72 h under N_2_ atmosphere, the reaction mixtures were cooled to room temperature and concentrated by rotary evaporation to obtain diversity-enhanced extracts. The diversity-enhanced extracts were submitted to RP-LCMS analysis and biological evaluation. The mixtures, in which any changes in either chemical structure or bioactivity after chemical diversification were observed, were repeated to check reproducibility.

Chemical diversification condition of 50% MeOH fraction with EtOH was optimized by varying concentration (0.2–1.0 mg/mL), temperature (25, 50, and 80 °C), and reaction time (24 h–7 days). The best reaction conditions were concentration 1 mg/3 mL, at 80 °C and for 72 h (Figure 2). The reactions of 1 mg of 50% MeOH fraction were conducted in 42 test tubes, then the reaction mixtures were pooled to obtain an overall 44 mg of diversity-enhanced extracts; from this, we succeeded in isolating the new product, after chemical treatment of substances present in NP extract, and obtained its ^1^H NMR data. The structure of this compound was identified to **1**, from matching it with the product obtained in the experiment in 3.6, which was developed later.

### 3.5. Larger-Scale Extraction, Prefractionation of the MeOH Crude Extract, and Purification of Naturally Occurring Compounds ***2**–**4***

To obtain sufficient quantity to determine the structure of **1** as well as to confirm that **1** was converted from **2** after the chemical reaction, larger-scale extraction of the sponge was conducted in the same procedure. The sponge (dry weight 203 g) was macerated in MeOH (1.5 L × 7 times) to obtain a crude MeOH extract (28 g). Prefractionation of the MeOH extract using RP-MPLC which was eluted with 50%MeOH in H_2_O provided 0.8 g of 50% MeOH fraction. Subsequently, the 50% MeOH fraction was purified by RP-MPLC, which was eluted with 20% MeOH in 0.1% TFA·H_2_O to afford **2** (221 mg) and **3** (23 mg). Furthermore, isolation of the 50% MeOH fraction by RP-MPLC which was eluted with 90% MeOH in 0.1% TFA·H_2_O also gave **4** (323 mg).

### 3.6. Chemical Conversion of **2** with Heat-Treated EtOH and Purification of ***1***

The chemical conversion of **2** in EtOH was gradually scaled up from 1 mg to 40 mg (conc. 1 mg/3 mL). The reaction flask was placed in an oil bath at 80 °C, and the mixture was heated for 72 h under N_2_ atmosphere. We examined the production of **1** by RP-LCMS in every gradual scaling up steps to ensure that the reactions proceeded well without any trouble. Then, the reaction mixtures were pooled (overall 106 mg) before purification.

The combined reaction mixture (106 mg) from **2** was purified by RP-MPLC which was eluted with 25% MeOH in 0.1% TFA·H_2_O to obtain **1** (19.1 mg, 18% yield). The structure and relative configuration of **1** were analyzed based on 1D- and 2D-NMR (Table 1, Figure 3, and Figures S3–S10), mass spectrometry (Figure S1), UV, IR (Figure S2), specific rotation value, chiral HPLC chromatogram (Figure S11), and comparison of spectroscopic data with **2** in the literature [18].

### 3.7. Experiments to Investigate the Possible Reaction Mechanisms

Compound **3** (1 mg) or **4** (1 mg) was dissolved in EtOH (3 mL) and heated at 80 °C for 72 h under N_2_ atmosphere. The reaction mixtures were analyzed by RP-LCMS.

To clarify whether the reaction proceeds through a radical mechanism, chemical conversions of **2** with heat-treated EtOH were conducted in the presence of galvinoxyl free radical (Tokyo Chemical Industry, Tokyo, Japan). Compound **2** (1 mg) was dissolved in EtOH (3 mL) in the presence and absence of 5% w/w galvinoxyl and then heated at 80 °C for 72 h under N_2_ atmosphere. The reaction mixtures were analyzed by RP-LCMS (Figure S12).

To study an effect of temperature on the production of **1**, chemical conversions of **2** with EtOH were conducted at various temperatures. Compound **2** (1 mg) was dissolved in EtOH (3 mL) and then heated at 25, 50, and 80 °C for 72 h under N_2_ atmosphere. The reaction mixtures were analyzed by RP-HPLC (Figure S13).

Moreover, chemical conversions of **2** (1 mg) with various aliphatic alcohols (3 mL), including MeOH, EtOH, isopropanol, and *n*-propanol, at their corresponding boiling temperatures were conducted for 72 h under N_2_ atmosphere. The reaction mixtures were analyzed by RP-LCMS (Scheme 3).

### 3.8. Anticancer Assay

The antiproliferative activity against 4 cancer cell lines was investigated by a WST-8 based assay. Human Jurkat leukemia and HT-29 colon cells were cultured in Roswell Park Memorial Institute (RPMI)-1640 medium (Nacalai tesque, Kyoto, Japan) and Hyclone^TM^ McCoy’s 5A medium (Cytiva, MA, USA), respectively, while PANC-1 pancreas and HeLa cervical cells were incubated in Dulbecco’s Modified Eagle’s Medium (DMEM, Nacalai tesque). All culture media contained 10% fetal bovine serum (FBS, Equitech-Bio Inc., TX, USA) and 50 μg/mL kanamycin (Fujifilm Wako Pure Chemical Corporation, Osaka, Japan). Each cell line was plated in a 96-well flat bottom plate at a concentration of 2000 cells/100 μL/well and treated with serially diluted compound for 4 days. Compounds to be tested were dissolved in DMSO for stock solution (20 mM) and freshly diluted into the corresponding medium so that the final concentration of DMSO was not more than 0.5%. Cells were incubated in a humidified incubator at 37 °C in an atmosphere of 5% CO_2_, then incubation with WST-8 (Nacalai tesque) up to 4 h. The absorbance of the formazan products was measured at 450 nm using an iMark^TM^ microplate reader (Bio-Rad, CA, USA). The mean IC_50_ values ± standard deviations (SD) were obtained from three independent experiments. The IC_50_ value of each experiment was determined using GraphPad Prism software. Cisplatin (Fujifilm Wako Pure Chemical Corporation) was used as a positive control.

### 3.9. Antimycobacterial Assay

The MIC of the tested compounds was determined by 3-(4,5-Dimethylthiazol-2-yl)-2,5-diphenyltetrazolium bromide (MTT) assay. *Mycobacterium smegmatis* and *M. bovis* BCG grew in the corresponding 7H9 broth under aerobic condition. *M. smegmatis* and *M. bovis* BCG were inoculated into a 96-well flat bottom plate at concentrations of 10^5^ and 10^6^ CFU/mL, respectively, and treated with serially diluted compound. Stock solutions of the compound in DMSO (20 mM) were diluted into the corresponding broth so that the final concentration of DMSO was not more than 0.5%. After incubation of *M. smegmatis* and *M. bovis* BCG at 37 °C for 1 and 6 days, respectively, 10 μL of MTT solution (10 mg/mL) was added to each well and further incubation was continued at 37 °C at least 12 h. The MTT formazan products were solubilized with the 50 μL of sodium dodecyl sulphate (SDS)-dimethylformamide (DMF) solution (10% SDS in 25% DMF·H_2_O), then the plate was placed at room temperature for 2–4 h. The optical density was measured at 560 nm using the SpectraMax^®^ M5 Microplate Reader (Molecular Devices, CA, USA). Isoniazid (Sigma-Aldrich, MO, USA) was used as a positive control.

## 4. Conclusions

To create and discover new chemical conversion and a new unnatural compound from NP extract, we first prepared a crude MeOH extract of marine sponge *Petrosia* (*Strongylophora*) sp. and prefractionated it by silica-gel (ODS) column chromatography. Secondly, the crude extract was chemically diversified by the selected solvent and optimized chemical conditions. Thirdly, the diversity-enhanced extract was analyzed by RP-LCMS. A new compound after chemical diversification was distinguished from the original NP extract. Lastly, the new unnatural component was scaled up, purified by column chromatography, and identified by various spectroscopy techniques. This study has provided a simple and rapid chemical diversification of the substances present in the NP extract with heat-treated EtOH which produced a new, unexpected derivative of alkaloid **1** from naturally occurring alkaloid **2** via alkoxylation at C-6. The reaction mechanism has not been clarified; however, the results suggest that a nitrogen of 3-carbamoylpyrrole ring A might be important for introduction of the angular ethoxy group, and the preparation of **1** was markedly sensitive to temperature. Moreover, two known natural compounds (**3** and **4**) were identified. None of them have been previously reported from this genus.

## Figures and Tables

**Table 1:** ^1^H NMR, ^13^C NMR data, and HMBC correlation of *rac*-6-*O*Et-cylindradine A (**1**) in DMSO-*d*_6_.

Position	*δ*_H_ (Multiplicity)	*δ*_C_ (Type)	HMBC Correlations from H to C
1 NH	13.46 (1H, s)		C-2, C-3, C-4, C-5
2		106.6 (C)	
3		95.5 (C)	
4		114.0 (C)	
5		131.5 (C)	
6		86.7 (C)	
7 NH	10.03 (s)		C-6, C-8
8		156.0 (C)	
9 NH	9.52 (s)		C-8, C-10
10		86.0 (C)	
11	2.07 (1H, m) 2.27 (1H, m)	34.3 (CH_2_)	C-10, C-12, C-13
12	2.04 (2H, m)	19.2 (CH_2_)	C-10, C-11, C-13
13	3.46 (1H, m) 3.57 (1H, m)	44.3 (CH_2_)	C-10, C-11, C-12
14 N			
15		157.2 (C)	
16 NH_2_	8.46 (s)		-
17	3.29 (1H, dq, *J* = 8.7, 7.0) 3.38 (1H, dq, *J* = 8.7, 7.0)	61.3 (CH_2_)	C-6, C-18
18	1.10 (3H, t, *J* = 7.0 Hz)	14.8 (CH_3_)	C-17

**Table 2:** The anticancer and antimycobacterial activities of **1**–**4**.

Compound	Anticancer Activity IC_50_ ± SD (μM)				Antimycobacterial Activity MIC (μM)	
	Jurkat	HT-29	PANC-1	HeLa	*Mycobacterium* *smegmatis*	*M. bovis*
**1**	>100	>100	>100	>100	>100	>100
**2**						
**3**	53 ± 8					
**4**	87 ± 9				50	
cisplatin	0.2 ± 0.02	-	-	-	-	
isoniazid	-				3.1	0.1

IC_50_: 50% inhibitory concentration. MIC: minimum inhibitory concentration. Jurkat: human leukemia cell line. HT-29: human colon cell line. PANC-1: human pancreas cell line. HeLa: human cervical cell line.

## Data Availability

Samples of the compounds are available from the corresponding author.

## Supplementary Materials

The following supporting information can be downloaded at: https://www.mdpi.com/article/10.3390/molecules28010143/s1, Figure S1–S10: HRMS, IR, ^1^H, ^13^C, COSY, HSQC, HMBC, and 2D-NOESY spectra of **1** in DMSO-*d*_6_ and/or CD_3_OD, Figure S11: Chiral HPLC chromatogram of **1**, Figure S12: RP-LCMS base peak chromatogram after heating of **2** with EtOH and presence/absence of 5%galvinoxyl, Figure S13: RP-HPLC chromatogram after heating of **2** with EtOH at various temperature, Physical and spectral data of **2**–**4**. Citation: [18,19,20,21,22,24].

## References

[1] Newman D.J., Cragg G.M. (2020). Natural products as sources of new drugs over the nearly four decades from 01/1981 to 09/2019. J. Nat. Prod..

[2] Butler M.S., Robertson A.A., Cooper M.A. (2014). Natural product and natural product derived drugs in clinical trials. Nat. Prod. Rep..

[3] Atanasov A.G., Zotchev S.B., Dirsch V.M., Supuran C.T., The International Natural Product Sciences Taskforce (2021). Natural products in drug discovery: Advances and opportunities. Nat. Rev. Drug Discov..

[4] Li G., Lou H.X. (2018). Strategies to diversify natural products for drug discovery. Med. Res. Rev..

[5] Lopez S.N., Ramallo I.A., Sierra M.G., Zacchino S.A., Furlan R.L. (2007). Chemically engineered extracts as an alternative source of bioactive natural product-like compounds. Proc. Natl. Acad. Sci. USA.

[6] Wu T., Jiang C., Wang L., Morris-Natschke S.L., Miao H., Gu L., Xu J., Lee K.H., Gu Q. (2015). 3,5-Diarylpyrazole derivatives obtained by ammonolysis of the total flavonoids from *Chrysanthemum indicum* extract show potential for the treatment of Alzheimer’s disease. J. Nat. Prod..

[7] Kawamura T., Matsubara K., Otaka H., Tashiro E., Shindo K., Yanagita R.C., Irie K., Imoto M. (2011). Generation of ‘unnatural natural product’ library and identification of a small molecule inhibitor of XIAP. Bioorg. Med. Chem..

[8] Masuda T., Nojima S., Miura Y., Honda S., Masuda A. (2015). An oxidative coupling product of luteolin with cysteine ester and its enhanced inhibitory activity for xanthine oxidase. Bioorg. Med. Chem. Lett..

[9] Salazar M.O., Micheloni O., Escalante A.M., Furlan R.L. (2011). Discovery of a beta-glucosidase inhibitor from a chemically engineered extract prepared through sulfonylation. Mol. Divers..

[10] Mendez L., Salazar M.O., Ramallo I.A., Furlan R.L. (2011). Brominated extracts as source of bioactive compounds. ACS Comb. Sci.

[11] Garcia P., Salazar M.O., Ramallo I.A., Furlan R.L. (2016). A new fluorinated tyrosinase inhibitor from a chemically engineered essential oil. ACS Comb. Sci..

[12] Kikuchi H., Sakurai K., Oshima Y. (2014). Development of diversity-enhanced extracts of *Curcuma zedoaria* and their new sesquiterpene-like compounds. Org. Lett..

[13] Kikuchi H., Ichinohe K., Kida S., Murase S., Yamada O., Oshima Y. (2016). Monoterpene indole alkaloid-like compounds based on diversity-enhanced extracts of iridoid-containing plants and their immune checkpoint inhibitory activity. Org. Lett..

[14] Kikuchi H., Kawai K., Nakashiro Y., Yonezawa T., Kawaji K., Kodama E.N., Oshima Y. (2019). Construction of a meroterpenoid-like compounds library based on diversity-enhanced extracts. Chem. Eur. J..

[15] Carroll A.R., Copp B.R., Davis R.A., Keyzers R.A., Prinsep M.R. (2021). Marine natural products. Nat. Prod. Rep..

[16] Haque N., Parveen S., Tang T., Wei J., Huang Z. (2022). Marine natural products in clinical use. Mar. Drugs.

[17] Lee Y.J., Cho Y., Tran H.N.K. (2021). Secondary metabolites from the marine sponges of the genus *Petrosia*: A literature review of 43 years of research. Mar. Drugs.

[18] Kuramoto M., Miyake N., Ishimaru Y., Ono N., Uno H. (2008). Cylindradines A and B: Novel bromopyrrole alkaloids from the marine sponge *Axinella cylindratus*. Org. Lett..

[19] Tasdemir D., Topaloglu B., Perozzo R., Brun R., O’Neill R., Carballeira N.M., Zhang X., Tonge P.J., Linden A., Ruedi P. (2007). Marine natural products from the Turkish sponge *Agelas oroides* that inhibit the enoyl reductases from *Plasmodium falciparum*, *Mycobacterium tuberculosis* and *Escherichia coli*. Bioorg. Med. Chem..

[20] Meyer S.W., Köck M. (2008). NMR studies of phakellins and isophakellins. J. Nat. Prod..

[21] Sharma G.M., Burkholder P.R. (1971). Structure of dibromophakellin, a new bromine-containing alkaloid from the marine sponge *Phakellia flabellata*. J. Chem. Soc. D.

[22] Forenza S., Minale L., Riccio R., Fattorusso E. (1971). New bromo-pyrrole derivatives from the sponge *Agelas oroides*. J. Chem. Soc. D.

[23] Lindel T. (2017). Chemistry and biology of the pyrrole-imidazole alkaloids. Alkaloids Chem. Biol..

[24] Iwata M., Kanoh K., Imaoka T., Nagasawa K. (2014). Total synthesis of (+)-cylindradine A. Chem. Commun..

[25] Lopes N., Ray S., Espada S.F., Bomfim W.A., Ray B., Faccin-Galhardi L.C., Linhares R.E.C., Nozawa C. (2017). Green seaweed *Enteromorpha compressa* (Chlorophyta, Ulvaceae) derived sulphated polysaccharides inhibit herpes simplex virus. Int. J. Biol. Macromol..

[26] Kamauchi H., Noji M., Kinoshita K., Takanami T., Koyama K. (2018). Coumarins with an unprecedented tetracyclic skeleton and coumarin dimers from chemically engineered extracts of a marine-derived fungus. Tetrahedron.

[27] Du Y., Sun J., Gong Q., Wang Y., Fu P., Zhu W. (2018). New alpha-pyridones with quorum-sensing inhibitory activity from diversity-enhanced extracts of a *Streptomyces* sp. derived from marine algae. J. Agric. Food Chem..

