# Supplementary material for: A New Tetracyclic Bromopyrrole-Imidazole Derivative through Direct Chemical Diversification of Substances Present in Natural Product Extract from Marine Sponge Petrosia (Strongylophora) sp
Source: Molecules. 2022 Dec 24;28(1):143. doi: 10.3390/molecules28010143 (PMC9821877; doi:10.3390/molecules28010143)
Supplement: Supplementary file 1 [file molecules-28-00143-s001.zip › molecules-2074933-supplementary.pdf]

## Supplementary Data

# A new tetracyclic bromopyrrole-imidazole derivative through direct chemical diversification of substances present in natural product extract from marine sponge *Petrosia* (*Strongylophora*) sp.

**Natchanun Sirimangkalakitti, Kazuo Harada, Makito Yamada, Masayoshi Arai, Mitsuhiro Arisawa**

Graduate School of Pharmaceutical Sciences, Osaka University, 1-6 Yamadaoka, Suita, Osaka 565-0871, Japan;
araim@phs.osaka-u.ac.jp (Ma.A.); arisaw@phs.osaka-u.ac.jp (Mi.A.)

**List of supporting information**

**Figure S1**. High-resolution MALDI mass spectrum of *rac*-6-*O*Et-cylindradine A (**1**)
**Figure S2**. IR spectrum of **1** in MeOH
**Figure S3**. $^{1}$H NMR spectrum (600 MHz) of **1** in DMSO-$d_6$
**Figure S4**. $^{1}$H NMR spectrum (600 MHz) of **1** in $CD_3OD$
**Figure S5**. $^{13}$C NMR spectrum (150 MHz) of **1** in DMSO-$d_6$
**Figure S6**. $^{13}$C NMR spectrum (150 MHz) of **1** in $CD_3OD$
**Figure S7**. $^{1}$H-$^{1}$H COSY spectrum (600 MHz) of **1** in DMSO-$d_6$
**Figure S8**. HSQC spectrum (600 MHz) of **1** in DMSO-$d_6$
**Figure S9**. HMBC spectrum (600 MHz) of **1** in DMSO-$d_6$
**Figure S10**. 2D-NOESY spectrum (600 MHz) of **1** in $CD_3OD$
**Figure S11**. Chiral HPLC chromatogram of **1**

**Figure S12**. RP-LCMS base peak chromatogram after heating of (-)-cylindradine A (**2**) with EtOH and presence/absence of 5% w/w galvinoxyl
**Figure S13**. RP-HPLC chromatogram after heating of **2** with EtOH at various temperatures

Physical and spectral data of **2**
Physical and spectral data of (-)-dibromophakellin (**3**)
Physical and spectral data of 4,5-dibromopyrrole-2-carboxylic acid (**4**)

**Figure S1**. High-resolution MALDI mass spectrum of *rac*-6-*O*Et-cylindradine A (**1**)

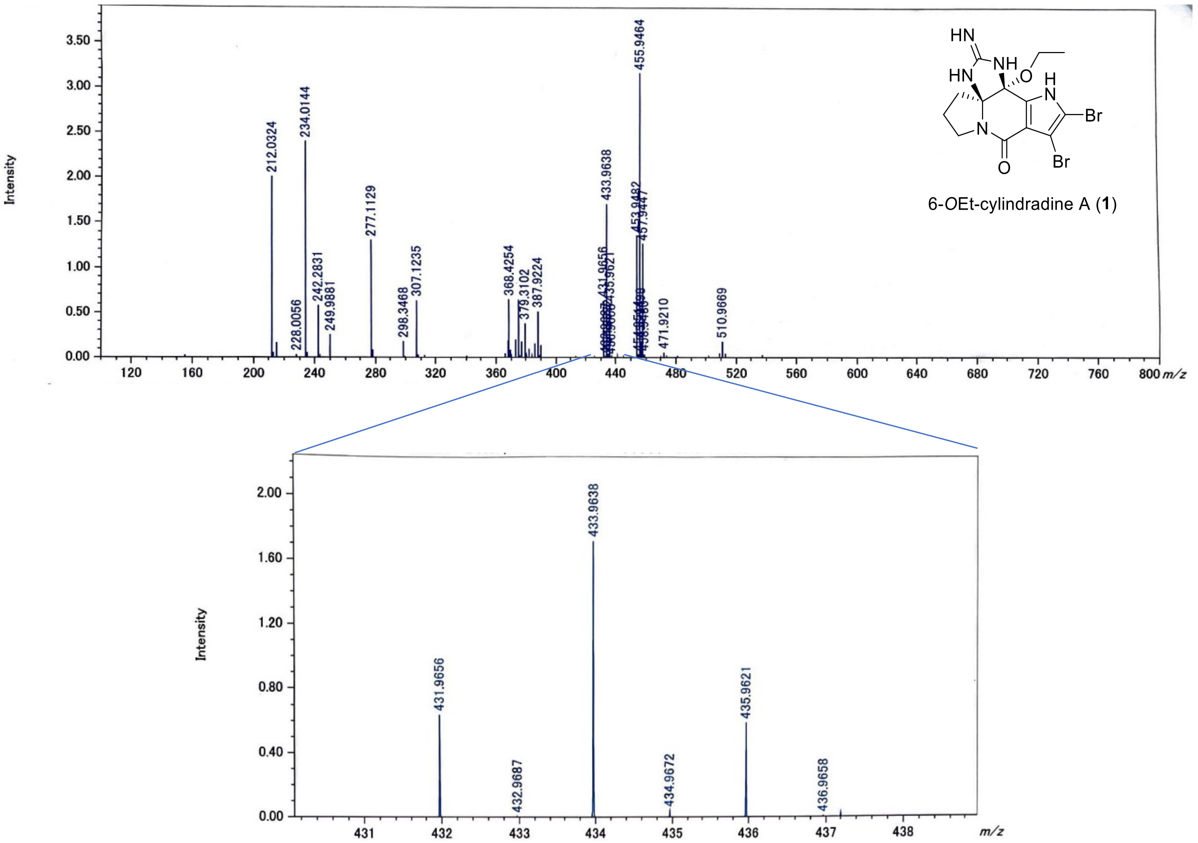


**Figure S2**. IR spectrum of *rac*-6-*O*Et-cylindradine A (**1**) in MeOH

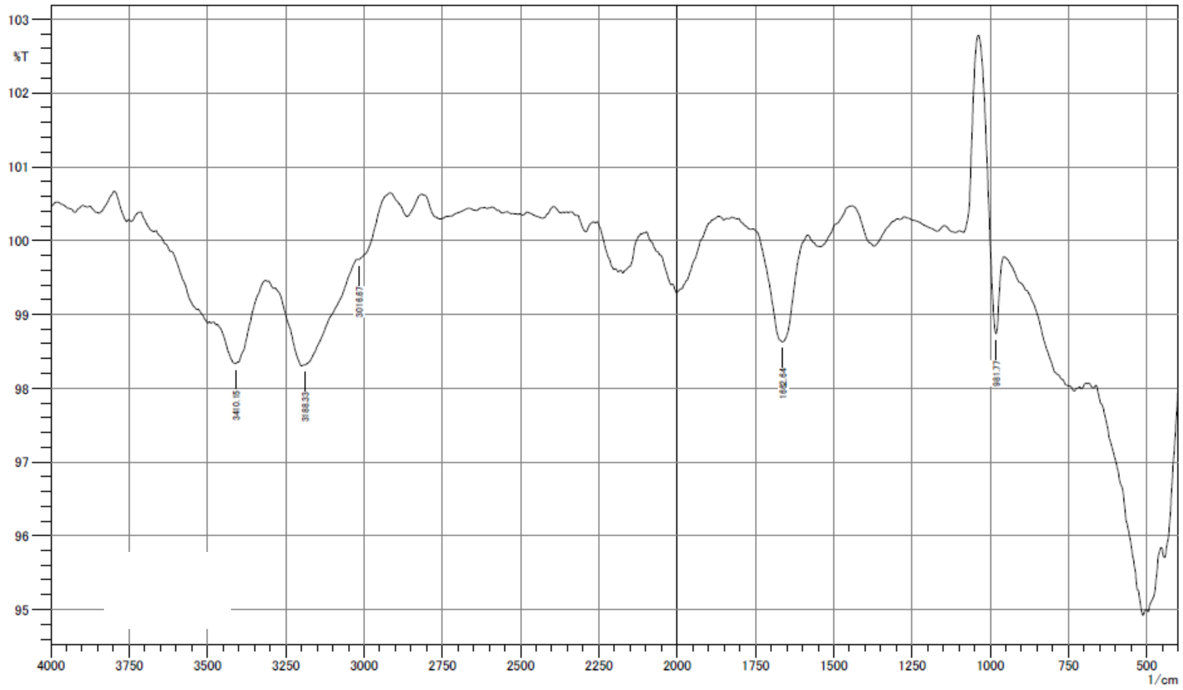

**Figure S3**. $^{1}$H NMR spectrum (600 MHz) of *rac*-6-*O*Et-cylindradine A (**1**) in DMSO-*d*$_6$

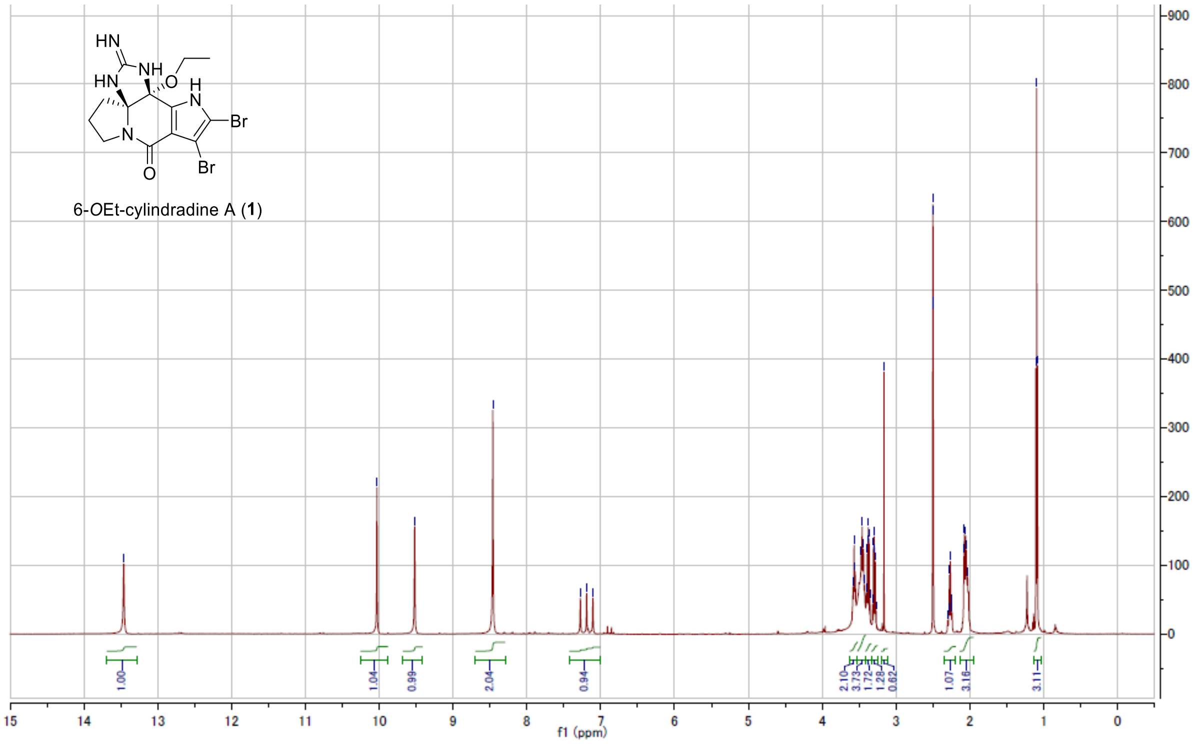


**Figure S4**. $^{1}$H NMR spectrum (600 MHz) of *rac*-6-*O*Et-cylindradine A (**1**) in $CD_3OD$

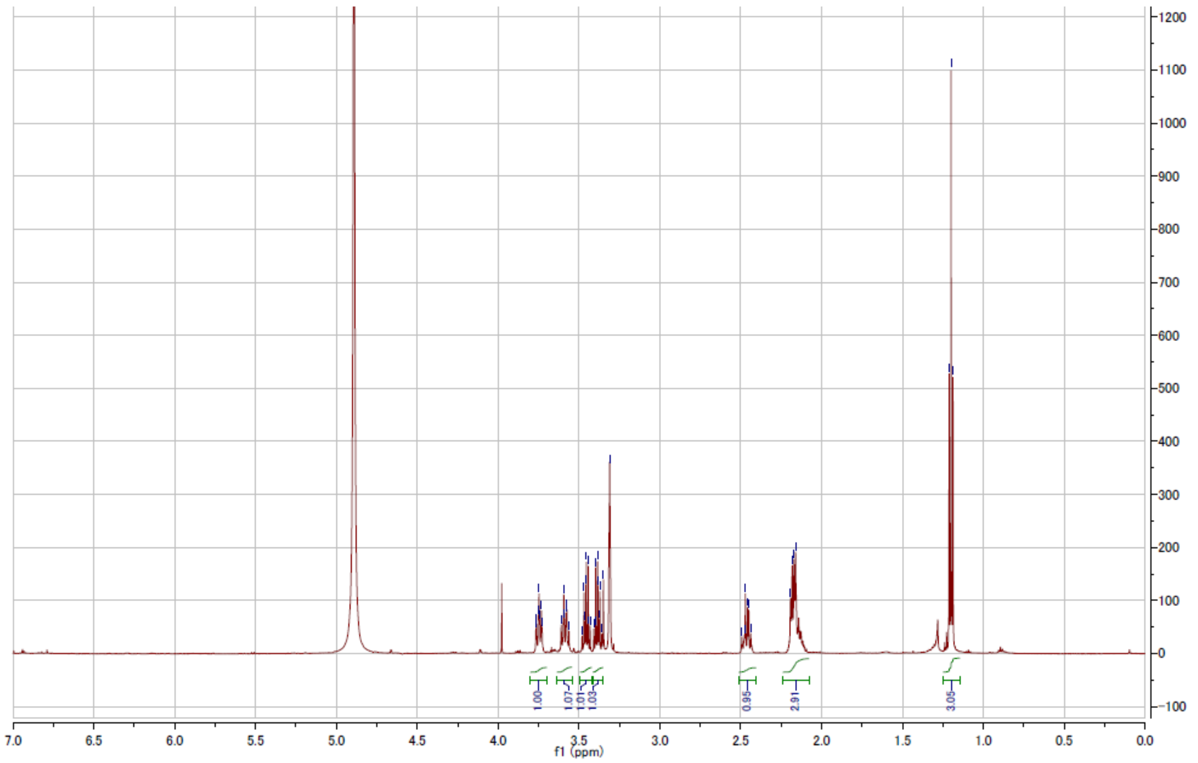

**Figure S5**. $^{13}$C NMR spectrum (150 MHz) of *rac*-6-*O*Et-cylindradine A (**1**) in DMSO-$d_6$

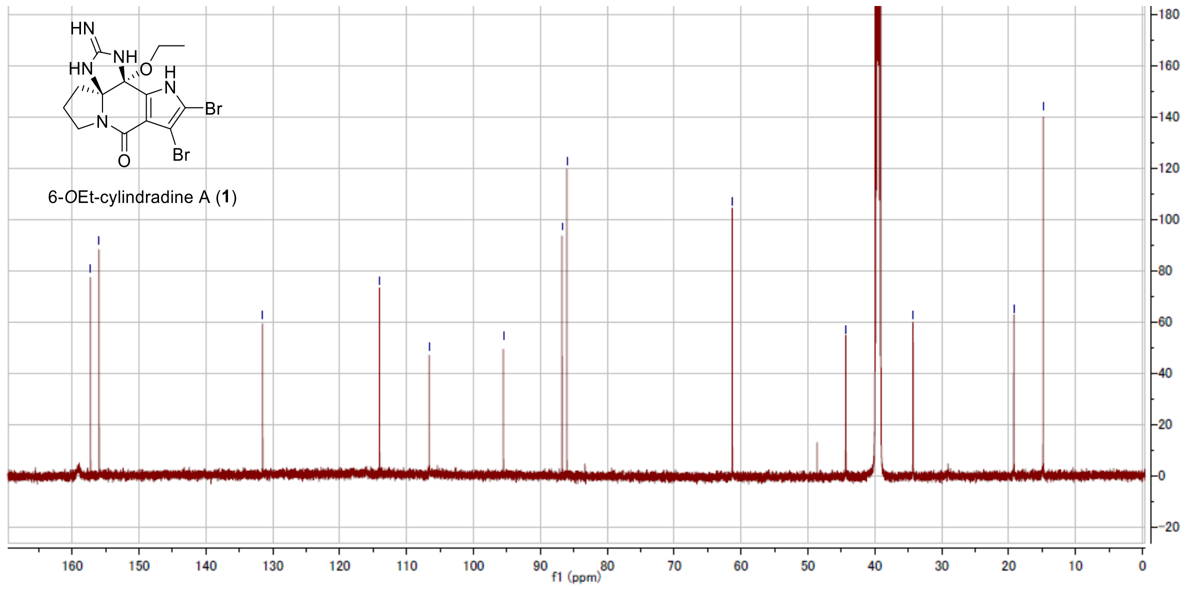


**Figure S6**. $^{13}$C NMR spectrum (150 MHz) of *rac*-6-*O*Et-cylindradine A (**1**) in $CD_3OD$

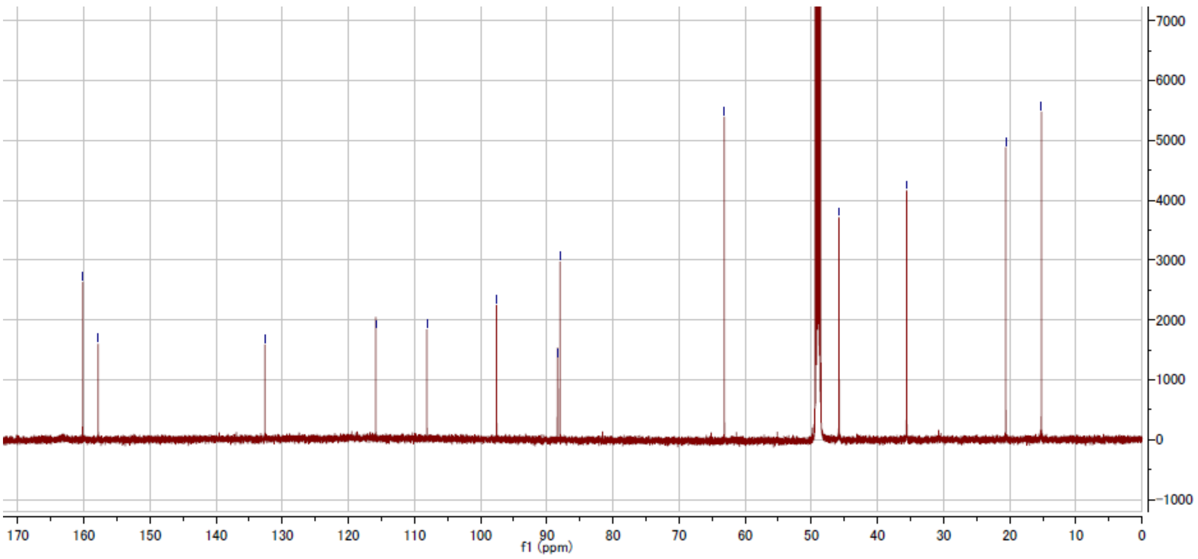

**Figure S7.** ¹H-¹H COSY spectrum (600 MHz) of *rac*-6-*O*Et-cylindradine A (**1**) in DMSO-$d_6$

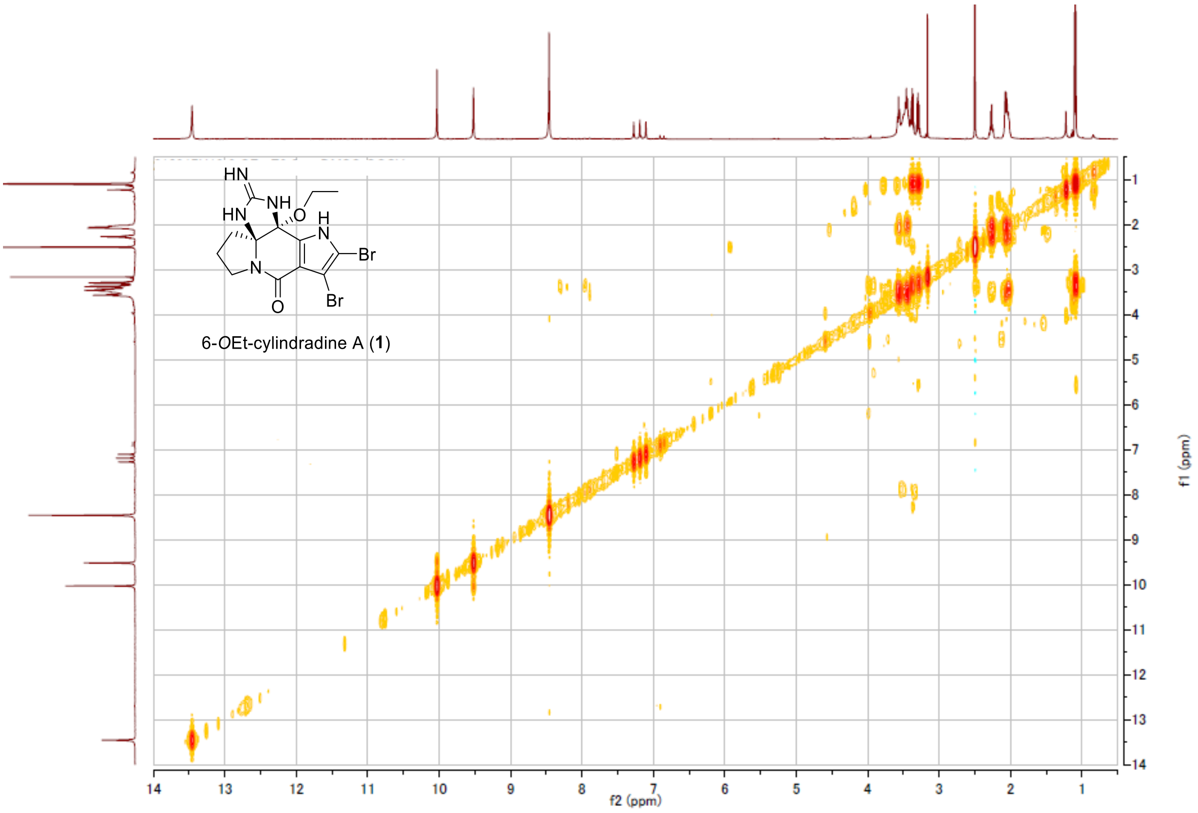


**Figure S8**. HSQC spectrum (600 MHz) of *rac*-6-*O*Et-cylindradine A (**1**) in DMSO-$d_6$

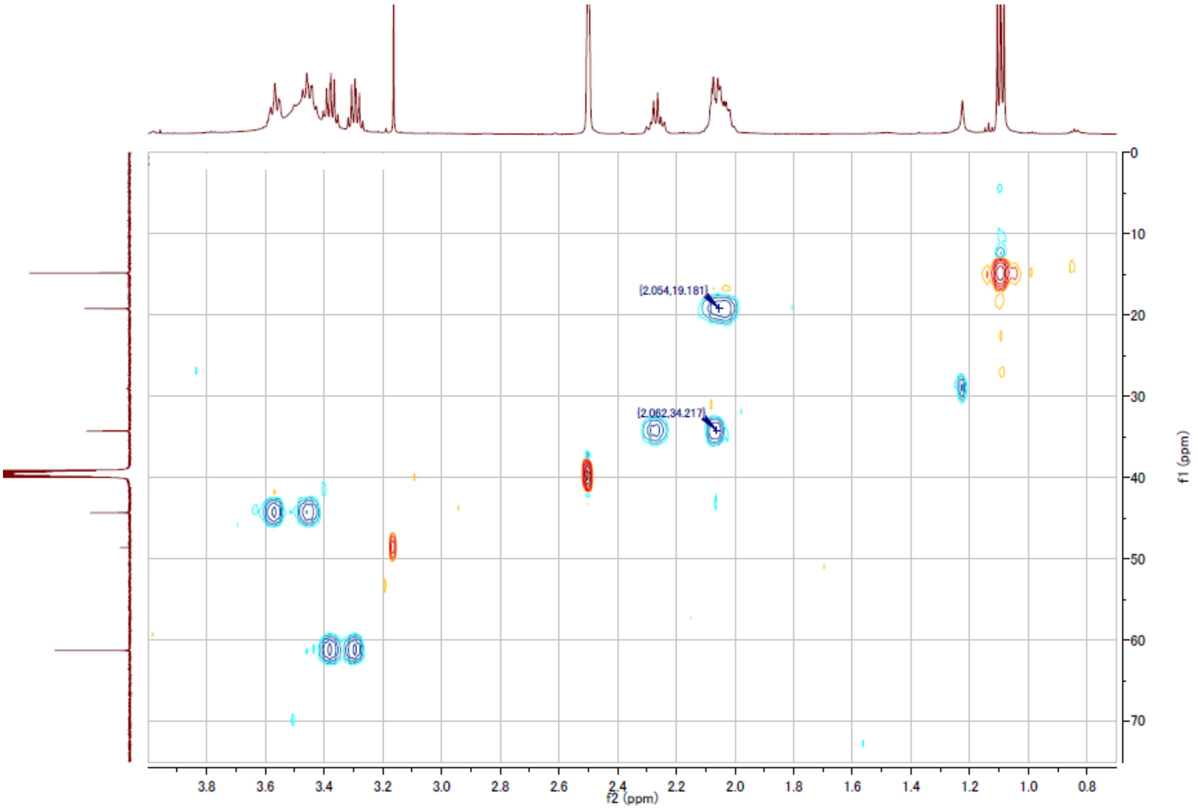

**Figure S9**. HMBC spectrum (600 MHz) of *rac*-6-*O*Et-cylindradine A (**1**) in DMSO-$d_6$

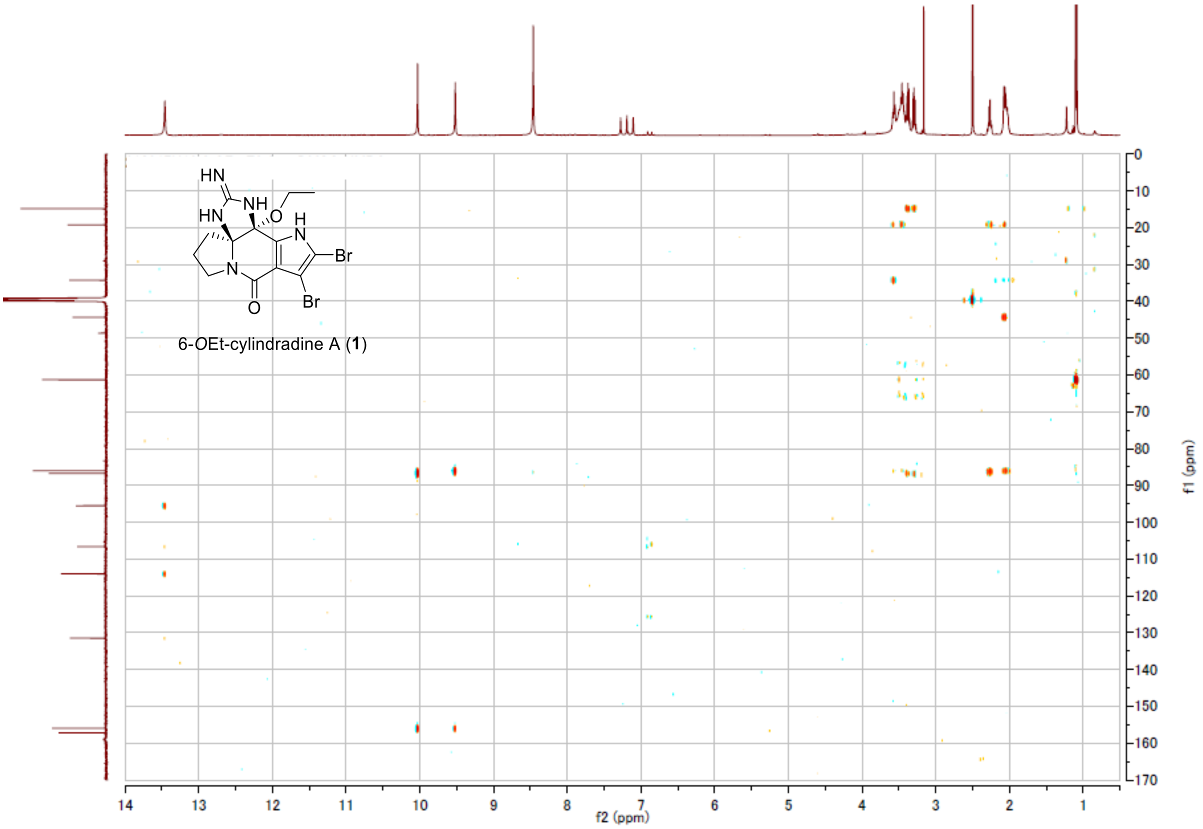


**Figure S10**. 2D-NOESY spectrum (600 MHz) of *rac*-6-*O*Et-cylindradine A (**1**) in $CD_3OD$

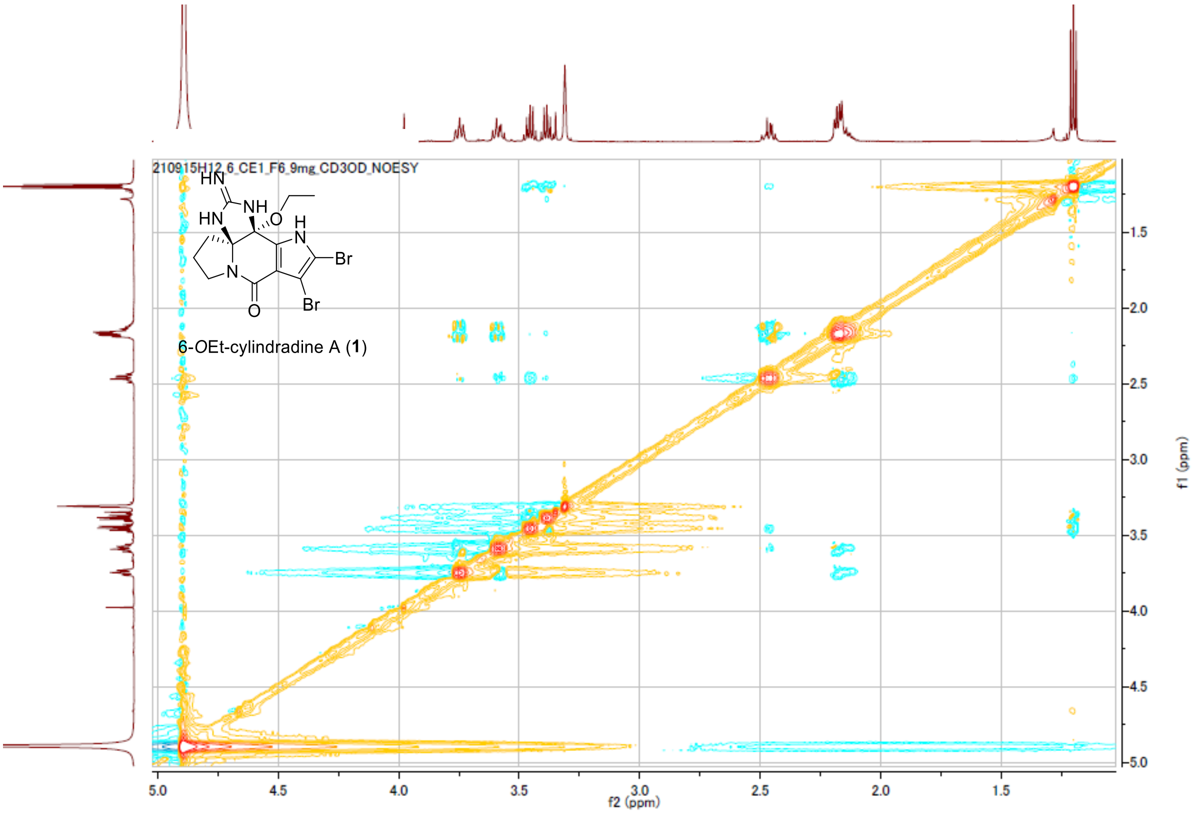

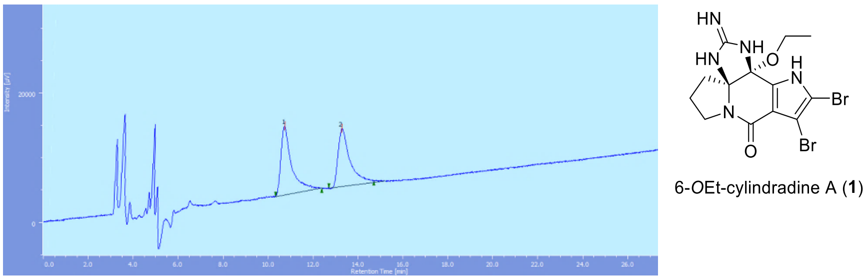


**Figure S11.** Chiral HPLC chromatograms of *rac*-6-*O*Et-cylindradine A (**1**). A HPLC system was composed of a PU-2089 Plus quaternary gradient pump, a CO-2065 Plus intelligent column oven, a MD-2010 Plus multiwavelength detector, and LC-NetII/ADC (Jasco Corporation, Japan). Chiral separation was performed on a CHIRALPAK® IE, 4.6×250 mm. The mobile phase was hexane:EtOH:diethylamine = 85:15:0.1, and flow rate was 1 mL/min at room temperature. The UV detection wavelength was set at 254 nm.

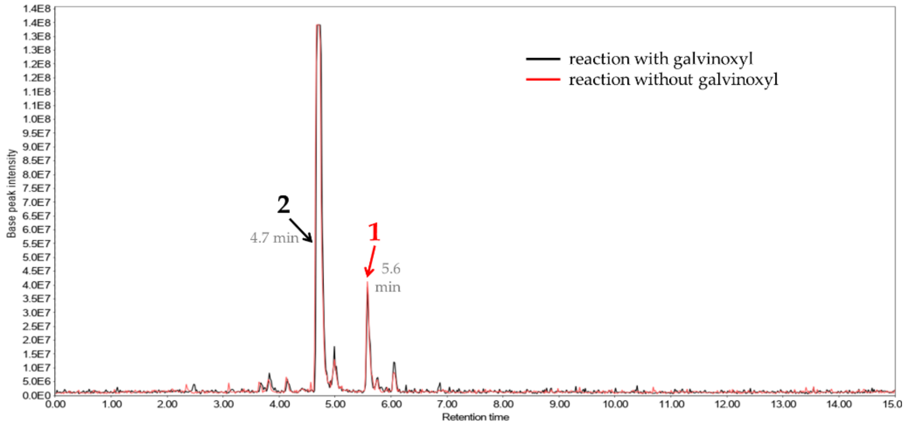


**Figure S12.** RP-LCMS base peak chromatogram (positive ion mode) after heating of (-)-cylindradine A (**2**) with EtOH and presence (black line) or absence of 5% w/w galvinoxyl (red line). Peaks of *rac*-6-*O*Et-cylindradine A (**1**) at 5.6 min and **2** at 4.7 min are pointed by red and black arrows, respectively.

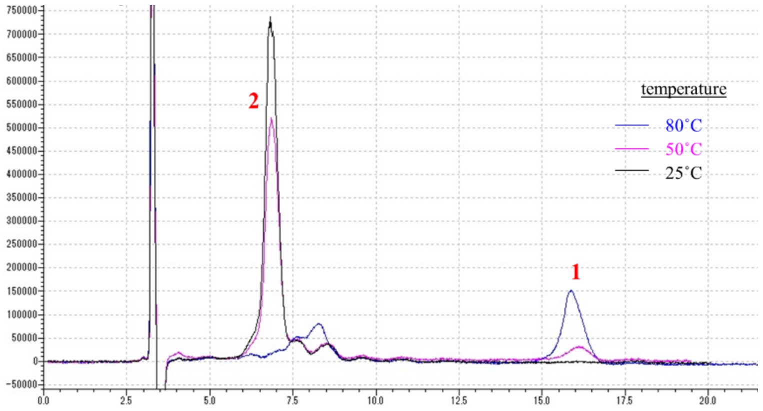


**Figure S13.** RP-HPLC chromatograms after heating of (-)-cylindradine A (**2**) with EtOH at various temperatures. The reactions of **2** were carried out at 25˚C (black line), 50˚C (pink line), and 80˚C (blue line) for 72 hours. HPLC analyses of **2** and *rac*-6-*O*Et-cylindradine A (**1**) were performed using XBridge C18 Column. The mobile phase was MeOH:water:formic acid = 35:65:0.1, and flow rate was 0.5 mL/min at 25˚C. The entire wavelength range of a PDA detector was set.

**Physical and spectral data of (-)-cylindradine A (2)**[1,2]

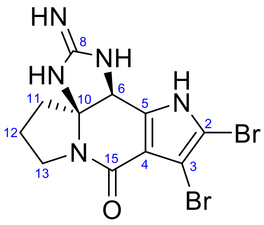

(-)-cylindradine A (**2**)

0.1% yield of sponge dry weight
white amorphous solid
m.p. 235 ˚C (decomp.) $[\alpha]_D^{20}$ -76.7 (c 0.22, MeOH); lit. $[\alpha]_D$ -75.3 (c 0.12, MeOH)[1]

$^1$H NMR (DMSO-$d_6$, 600 MHz) $\delta$ 5.21 (1H, s, H-6), 3.54 (1H, m, H-13a), 3.36 (overlapped, H-13b), 2.20 (2H, m, H-11), 2.03 and 1.97 (2H, m, H-12); $^{13}$C NMR (DMSO-$d_6$, 150 MHz) $\delta$ 158.1 (C-15), 156.6 (C-8), 111.1 (C-4), 82.8 (C-10), 54.2 (C-6), 44.3 ($CH_2$-13), 39.5 ($CH_2$-11), 19.2 ($CH_2$-12)

$^1$H NMR ($CD_3OD$, 600 MHz) $\delta$ 5.27 (1H, s, H-6), 3.73 (1H, ddd, *J* = 9.9, 7.4, 2.1 Hz, H-13a), 3.58 (ddd, *J* = 9.9, 7.0, 1.7 Hz, H-13b), 2.40 (1H, ddd, *J* = 10.6, 10.5, 6.3 Hz, H-11a), 2.29 (1H, dd, *J* = 10.5, 4.8 Hz, H-11b), 2.14 (2H, m, H-12);

$^{13}$C NMR ($CD_3OD$, 150 MHz) $\delta$ 158.7 (C-15), 156.7 (C-8), 132.1 (C-5), 111.3 (C-4), 104.6 (C-2), 95.7 (C-3), 83.1 (C-10), 53.8 (CH-6), 43.8 ($CH_2$-13), 38.9 ($CH_2$-11), 18.7 ($CH_2$-12)
HRMALDIMS *m/z* 387.9380, 389.9361, 391.9343 (1:2:1) $[M+H]^+$, calcd for $C_{11}H_{12}{}^{79}Br_2N_5O$ 387.9403; 409.9201, 411.9183, 413.9164 (1:2:1) [M+Na]+, calcd for $C_{11}H_{11}{}^{79}Br_2N_5NaO$ 409.9223
IR (NaCl) $\nu_{max}$ 3305, 3170, 2976, 1690, 1642, 1562, 1496, 1428, 1389, 1344, 1206, 1131, 1013, 969, 790, 764 cm$^{-1}$

**Physical and spectral data of (-)-dibromophakellin (3)**[3,4]

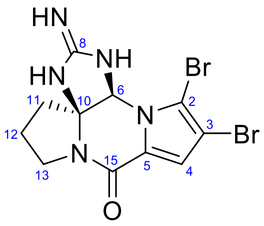

(-)-dibromophakellin (**3**)

0.01% yield of sponge dry weight
white amorphous solid
m.p. 230 ˚C (decomp.); lit. m.p. 237-245 ˚C (decomp.)[3]
$[\alpha]_D^{21}$ -111 (c 0.51, MeOH); lit. $[\alpha]_D^{25}$ -203 (c 0.12, MeOH)[3]
$^1$H NMR (DMSO-$d_6$, 600 MHz) $\delta$ 10.43, 9.93, 8.69, 8.32 (each 1H, each br, NH), 7.02 (1H, s, H-4), 6.31 (1H, s, H-6), 3.66 (1H, m, H-13a), 3.48 (1H, m, H-13b), 2.40 (1H, m, H-11a), 2.28 (1H, m, H-11b), 2.06 (2H, m, H-12); $^{13}$C NMR (DMSO-$d_6$, 150 MHz) $\delta$ 156.5 (C-15), 153.7 (C-8), 125.0 (C-5), 114.8 (C-4), 106.1 (C-2), 102.0 (C-3), 82.4 (C-10), 68.2 (CH-6), 44.7 ($CH_2$-13), 39.7 ($CH_2$-11), 19.0 ($CH_2$-12)
HRMALDIMS *m/z* 387.9396, 389.9376, 391.9358 (1:2:1) $[M+H]^+$, calcd for $C_{11}H_{12}{}^{79}Br_2N_5O$ 387.9403
IR (NaCl) $\nu_{max}$ 3172, 1672, 1561, 1441, 1386, 1204, 1139, 843, 801, 724 cm$^{-1}$

---

[1] Kuramoto, M.; Miyake, N.; Ishimaru, Y.; Ono, N.; Uno, H. Cylindradines A and B: Novel bromopyrrole alkaloids from the marine sponge *Axinella cylindratus*. *Org. Lett*. **2008**, *10*, 5465-5468.

[2] Iwata, M.; Kanoh, K.; Imaoka, T.; Nagasawa, K. Total synthesis of (+)-cylindradine A. *Chem. Commun.* **2014**, *50*, 6991-6994.

[3] Sharma, G.M.; Burkholder, P.R. Structure of dibromophakellin, a new bromine-containing alkaloid from the marine sponge *Phakellia flabellata*. *J. Chem. Soc. D* **1971**, 151-152.

[4] Meyer, S.W.; Köck, M. NMR studies of phakellins and isophakellins. *J. Nat. Prod.* **2008**, *71*, 1524-1529.

**Physical and spectral data of 4,5-dibromopyrrole-2-carboxylic acid (4)**[5,6]

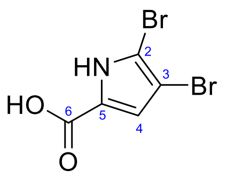

4,5-dibromopyrrole-
2-carboxylic acid (**4**)

0.2% yield of sponge dry weight
white amorphous solid
m.p. 185 ˚C (decomp.); lit. subl. 148 ˚C [5]
$^1$H NMR ($CD_3OD$, 600 MHz) *δ* 6.79 (1H, s, H-4); $^{13}$C NMR ($CD_3OD$, 150 MHz) *δ* 163.0 (C-6), 126.8 (C-2), 118.2 (CH-4), 107.0 (C-2), 100.3 (C-3)
$^1$H NMR (DMSO-$d_6$, 600 MHz) *δ* 6.78 (1H, s, H-4); $^{13}$C NMR (DMSO-$d_6$, 150 MHz) *δ* 160.8 (C-6), 126.2 (C-2), 116.3 (CH-4), 105.9 (C-2), 98.5 (C-3)
HRMALDIMS *m/z* 265.8460, 267.8508, 269.8456 (1:2:1) $[M+H]^+$, calcd for $C_5H_2{}^{79}Br_2NO_2$ 265.8458
IR (NaCl) $\nu_{max}$ 3406, 3131, 2977, 1681, 1555, 1430, 1373, 1312, 1199, 1023, 970, 835, 767 cm$^{-1}$

---

5 Forenza, S.; Minale, L.; Riccio, R.; Fattorusso, E. New bromo-pyrrole derivatives from the sponge *Agelas oroides*. *J. Chem. Soc. D* **1971**, 1129-1130.

6 Tasdemir, D.; Topaloglu, B.; Perozzo, R.; Brun, R.; O'Neill, R.; Carballeira, N.M.; Zhang, X.; Tonge, P.J.; Linden, A.; Ruedi, P. Marine natural products from the Turkish sponge *Agelas oroides* that inhibit the enoyl reductases from *Plasmodium falciparum*, *Mycobacterium tuberculosis* and *Escherichia coli*. *Bioorg. Med. Chem.* **2007**, *15*, 6834-6845.
